# Maternal feeding patterns affect the offspring’s brain: focus on serotonin 5-HT_2C_ and 5-HT_2A_ receptors

**DOI:** 10.1007/s43440-021-00298-0

**Published:** 2021-06-19

**Authors:** Kinga Gawlińska, Dawid Gawliński, Małgorzata Filip, Edmund Przegaliński

**Affiliations:** Department of Drug Addiction Pharmacology, Maj Institute of Pharmacology Polish Academy of Sciences, Smętna Street 12, 31-343 Kraków, Poland

**Keywords:** Fetal programming, High-fat diet, Maternal diet, Offspring brain, Serotonin 5-HT_2C_ receptor, Serotonin 5-HT_2A_ receptor

## Abstract

**Background:**

Recent studies have shown a relationship between the composition of the maternal diet and acquiring a risk of mental illnesses through changes in the offspring’s brain. This study assessed the role of a modified maternal diet on the levels of serotonin (5-HT)_2C_ and 5-HT_2A_ receptors in the offspring brain.

**Methods:**

Wistar rat dams during gestation and lactation were maintained either on a standard (SD) or special diets: high-fat (HFD), high-carbohydrate (rich in sucrose, HCD) or mixed (MD). Offspring were weaned to SD after lactation, and at postnatal days (PNDs) 28 and 63 changes in the 5-HT_2C_ and 5-HT_2A_ receptor levels were evaluated in their prefrontal cortex (PFCx), nucleus accumbens (NAc), dorsal striatum (DSTR) and hippocampus (HIP).

**Results:**

Maternal HFD reduced the expression of 5-HT_2C_ receptors in male rats at PND 28 in the PFCx, NAc, and DSTR but increased it at PND 63 in male animals in the NAc and DSTR. HCD induced a decrease in the expression of 5-HT_2C_ receptors in male offspring at PND 28 but increased it in female rats at PND 63 in the PFCx. MD reduced 5-HT_2C_ receptor expression in males at PND 28 in the PFCx and increased it in male and female offspring at PND 28 in the HIP. Moreover, maternal HFD reduced 5-HT_2A_ receptor levels within the PFCx in adolescent male offspring.

**Conclusion:**

Our findings indicate that a modified maternal diet induces age- and sex-specific adaptive changes mainly in 5-HT_2C_ receptors, which may contribute to disturbances in the offspring brain.

**Graphic abstract:**

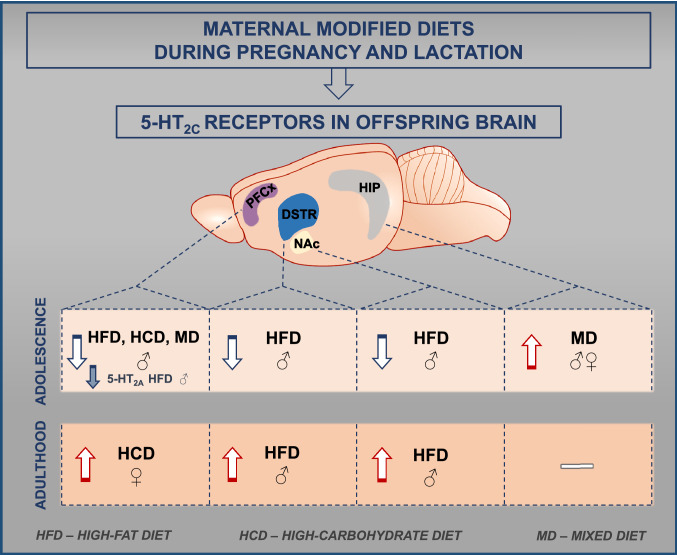

## Introduction

The global epidemic of obesity increasingly occurring among women of reproductive age and around conception is due to excessive food intake and an unhealthy lifestyle. In many countries, more than one-third of pregnant women are overweight or obese, which has numerous negative effects on the health of the offspring [1]. Currently, much evidence indicates that not only obesity but also maternal feeding patterns during pregnancy and lactation may be responsible for fetal developmental adaptations that permanently affect the offspring’s metabolism, neurodevelopment, and behavior throughout their life. This phenomenon is called “fetal programming” and could be associated with epigenetic changes during early life (see review [2]). Epidemiological and experimental studies suggest a link between early exposure to an unbalanced maternal diet (e.g., high-fat or high-carbohydrate) and susceptibility to morphological, molecular and functional changes in the offspring’s brain as well as neurodevelopmental or mental disorders, such as attention-deficit hyperactivity disorder (ADHD), autism spectrum disorder (ASD), anxiety, depression, schizophrenia and eating disorders [2–4].

Serotonin (5-hydroxytryptamine, 5-HT) is an important neurotransmitter in the central nervous system and is an endogenous ligand for seven types of 5-HT receptors (5-HT_1_–5-HT_7_). One of the interesting links to mental illness from the nutritional and pathological points of view are the 5-HT_2A_ and 5-HT_2C_ receptors, seven-transmembrane, G-protein linked receptors positively coupled to phospholipase C. 5-HT_2A_ receptors are involved in the regulation of learning and memory [5], and 5-HT_2C_ receptor signaling is related to physiological brain functions, such as regulation of appetite, food intake and energy balance, stress response or sleep regulation [6]. The 5-HT_2C_ receptor has structural similarity to the 5-HT_2A_ receptor, as well as similar pharmacological profiles and cellular signaling pathways [7]. Given the important role of 5-HT neurotransmission in brain development, function, and mental disorders, disturbances in 5-HT_2A_ and 5-HT_2C_ receptor levels resulting from abnormal conditions of intrauterine development may have critical implications for the behavior and mental health of offspring during their entire lifetime [8].

In light of the above information and recent results from our group showing that maternal modified diets limited to periods of pregnancy and lactation predispose offspring to the development of depressive-like behavior [9, 10] or changes in the strength of cocaine-seeking behavior [11, 12], in this study, we investigated the role of early exposure to maternal high-fat (HFD), high-carbohydrate (rich in sucrose, HCD) and a mixed diet (rich in fat and carbohydrate, MD) on 5-HT_2A_ and 5-HT_2C_ receptors profiles within the brain structures (prefrontal cortex, dorsal striatum, nucleus accumbens, and hippocampus) associated with mental disorders during offspring development (adolescence and adulthood).

## Materials and methods

### Animals and diets

This study was performed in accordance with the guidelines of the EU Directive 2010/63/EU with the approval of the Ethical Committee at the Maj Institute of Pharmacology Polish Academy of Sciences (approval number 1270/2015; December 17, 2015). Every effort was made to minimize suffering and the number of animals used.

Wistar rats from Charles River (Sulzfeld, Germany) were housed in standard cages in an animal room maintained at 22 ± 2 °C and 55 ± 10% humidity under a 12 h light–dark cycle (lights on at 6.00 a.m.). The animals had free access to water and food.

Female virgin rats (200–240 g), after the acclimatization period and during the proestrus phase (smears from females were assessed to determine the estrous cycle phase) were mated with males. The pregnancy was confirmed by examining the vaginal smears for the presence of sperm. Then, the pregnant females were individually housed and randomly assigned to four groups: standard diet (SD, 65% carbohydrate, 13% fat, 22% protein, 3.4 kcal/g; VRF1; Special Diets Services, Witham, UK) or modified diets purchased from Altromin (Lage, Germany): HFD (24% carbohydrate, 60% fat, 16% protein, 5.31 kcal/g; C1057 mod.), HCD (70% carbohydrate: rich in sucrose, 12% fat, 18% protein, 3.77 kcal/g; C1010) or MD (56% carbohydrate, 28% fat, 16% protein, 3.90 kcal/g; C1011). Dams were fed these diets ad libitum during pregnancy (21 days) and lactation (21 days). The special diets used in this study did not affect the litter size or birth weight of the offspring [11]. Litter sizes were normalized to 9–12 pups with a sex ratio as close to 1:1 as possible. To reduce “litter effects” [13], animals for each group were selected from 3–4 different dams. After weaning, offspring at postnatal (PND) 22 were separated according to sex and kept on a SD. Male and female offspring were used in this study. Biochemical tests were performed at PNDs 28 and 63.

### Brain tissue isolation

Groups of male and female offspring (*n* = 8 in each group) were sacrificed through decapitation at PNDs 28 and 63. The prefrontal cortex (including the infralimbic, prelimbic and cingulate cortices; bregma: 5.2–2.7 mm), nucleus accumbens, dorsal striatum, and hippocampus, were dissected according to The Rat Brain Atlas [14] and were immediately frozen on dry ice and stored at − 80 °C until analysis. All tissues were collected between 9.00 and 12.00 a.m.

### Enzyme-linked immunosorbent assay (ELISA)

Frozen brain structures were homogenized using a sonicator (EpiShear™ Probe Sonicator; Active Motif, Carlsbad, CA, USA) in 10% (w/v) of 0.32 M sucrose HEPES buffer (containing 145 mM NaCl, 5 mM KCl, 2 mM CaCl_2_, 1 mM MgCl_2_, 5 mM glucose and 5 mM HEPES) with a protease inhibitor cocktail (Complete, Roche Diagnostics, Mannheim, Germany). Isolation of the synaptosomal fraction was performed to observe molecular changes in the protein composition at the synapses and to determine the 5-HT_2C_ and 5-HT_2A_ receptor concentrations as described previously [12]. For protein determination, a bicinchoninic acid (BCA) protein assay kit (Pierce™ BCA Protein Assay Kit, Thermo Scientific, Rockford, IL, USA) was used. The concentrations of 5-HT_2C_ and 5-HT_2A_ receptors in the selected brain structures were measured using ELISA (Cat. No E1793Ra, Cat. No E1799Ra; BT LAB, Shanghai, China) according to the manufacturer’s instructions. Forty microliters of each sample (*n* = 8 for the prefrontal cortex and nucleus accumbens; *n* = 7 for the dorsal striatum and hippocampus) were transferred to 96-well ELISA plates in duplicate with standards. The absorbance was measured at a wavelength (*λ*) of 450 nm using a Multiskan Spectrum spectrophotometer (Thermo Labsystems, Philadelphia, PA, USA). The concentrations of the 5-HT_2C_ and 5-HT_2A_ receptors were calculated from a standard curve and are expressed as ng/mg of protein and pg/mg of protein, respectively. The precision was intra-assay: CV < 8% and interassay: CV < 10%.

### Statistical analyses

Statistical analyses were performed using GraphPad Prism 9.1.0 software (GraphPad Software, San Diego, CA, USA). All data are expressed as the mean (± SEM). Statistical analysis was performed using a two-way analysis of variance (ANOVA, diet × sex). Post hoc Tukey’s tests were used to examine differences between group means. *p* < 0.05 was considered statistically significant.

## Results

### Adolescence

**Fig. 1:**
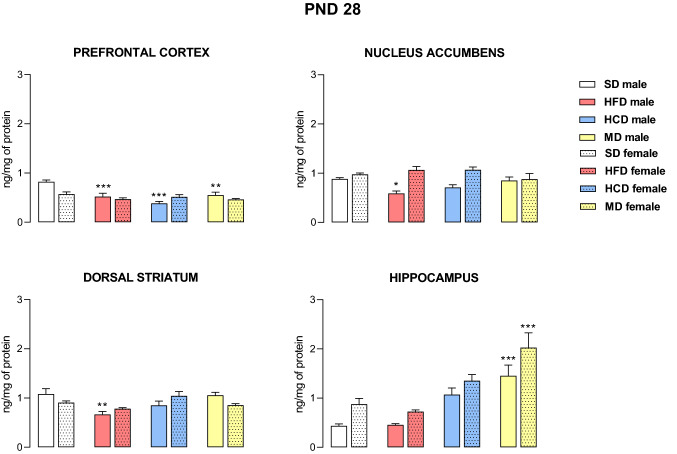
The levels of the 5-HT_2C_ receptor in the prefrontal cortex, nucleus accumbens, dorsal striatum and hippocampus of male and female offspring whose mothers were fed a high-fat diet (HFD), high-carbohydrate diet (HCD), mixed diet (MD) or standard diet (SD) at postnatal day (PND) 28. *n* = 8 rats/group for the prefrontal cortex and nucleus accumbens; *n* = 7 rats/group for the dorsal striatum and hippocampus). Significance was determined using two-way ANOVA followed Tukey’s post hoc test. *p < 0.05, **p < 0.01, ***p < 0.001 versus the corresponding SD group

The effects of modified maternal diets on 5-HT_2C_ receptor levels in the brains of male and female offspring at PND 28 are shown in Fig. 1. In adolescent offspring, two-way ANOVA indicated a significant effect of diet on 5-HT_2C_ receptor levels within the prefrontal cortex (F_3,56_ = 11.50, *p* < 0.001), dorsal striatum (F_3,48_ = 6.20, *p* < 0.01) and hippocampus (F_3,48_ = 24.39, *p* < 0.001) and a diet × sex interaction in the prefrontal cortex (F_3,56_ = 6.03, *p* < 0.01), dorsal striatum (F_3,48_ = 4.27, *p* < 0.01) and nucleus accumbens (F_3,56_ = 5.50, *p* < 0.01). Male offspring from the HFD group showed decreased 5-HT_2C_ receptor concentrations in the prefrontal cortex (*p* < 0.001), nucleus accumbens (*p* < 0.05) and dorsal striatum (*p* < 0.01), while maternal HCD and MD during pregnancy and lactation reduced 5-HT_2C_ receptor levels within the prefrontal cortex (*p* < 0.001 and *p* < 0.01, respectively). Moreover, upregulation of the hippocampal 5-HT_2C_ receptor was observed in both male and female offspring from the MD group (*p* < 0.001).

**Fig. 2:**
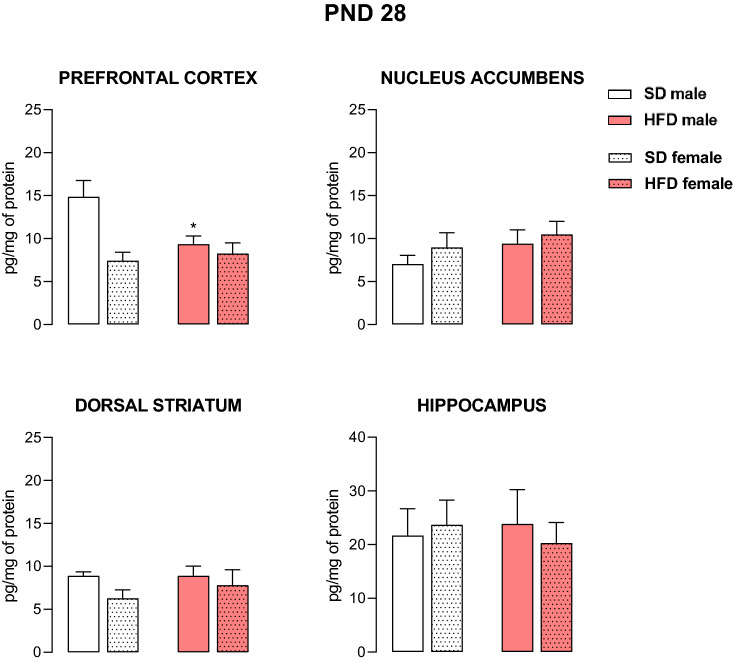
The levels of the 5-HT_2A_ receptor in the prefrontal cortex, nucleus accumbens, dorsal striatum and hippocampus of male and female offspring whose mothers were fed a high-fat diet (HFD) or standard diet (SD) at postnatal day (PND) 28. *n* = 8 rats/group for the prefrontal cortex and nucleus accumbens; *n* = 7 rats/group for the dorsal striatum and hippocampus. Significance was determined using two-way ANOVA followed Tukey’s post hoc test. *p < 0.05 versus the corresponding SD group

We determined the level of 5-HT_2A_ receptors in the HFD group, in which the most numerous changes in the levels of the 5-HT_2C_ receptors were determined. At PND 28, two-way ANOVA indicated a significant effect of diet × sex interaction on 5-HT_2A_ receptor levels only within the prefrontal cortex (F_1,28_ = 5.66, *p* < 0.05). In fact, a maternal HFD decreased the cortical 5-HT_2A_ receptor concentration in male offspring (*p* < 0.05) (Fig. 2).

### Adulthood

**Fig. 3:**
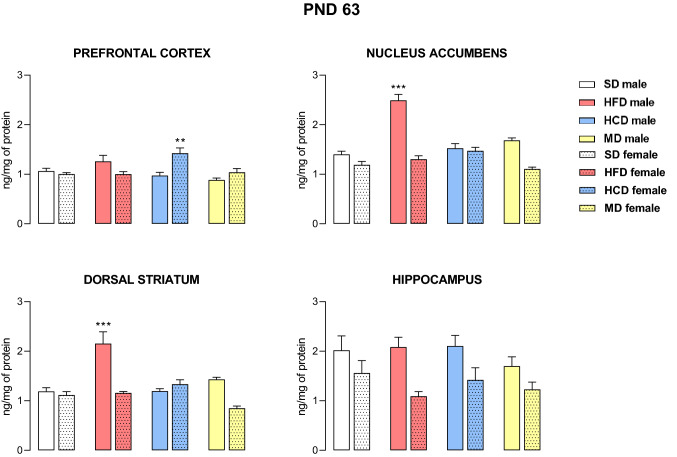
The levels of the 5-HT_2C_ receptor in the prefrontal cortex, nucleus accumbens, dorsal striatum and hippocampus of male and female offspring whose mothers were fed a high-fat diet (HFD), high-carbohydrate diet (HCD), mixed diet (MD) or standard diet (SD) at postnatal day (PND) 63. *n* = 8 rats/group for the prefrontal cortex and nucleus accumbens; *n* = 7 rats/group for the dorsal striatum and hippocampus. Significance was determined using two-way ANOVA followed Tukey’s post hoc test. **p < 0.01, ***p < 0.001 versus the corresponding SD group

**Fig. 4:**
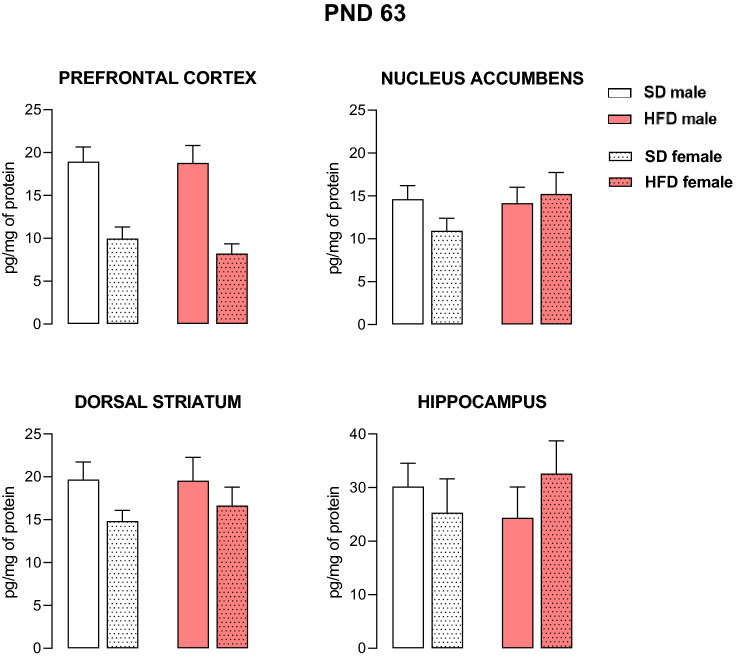
The levels of the 5-HT_2A_ receptor in the prefrontal cortex, nucleus accumbens, dorsal striatum and hippocampus of male and female offspring whose mothers were fed a high-fat diet (HFD) or standard diet (SD) at postnatal day (PND) 63. *n* = 8 rats/group for the prefrontal cortex and nucleus accumbens; *n* = 7 rats/group for the dorsal striatum and hippocampus. Significance was determined using two-way ANOVA followed Tukey’s post hoc test

In adult animals, statistical analysis also showed the importance of the dietary effect on the expression of the 5-HT_2C_ receptor in the prefrontal cortex (F_3,56_ = 3.78, *p* < 0.05), nucleus accumbens (F_3,56_ = 24.15, *p* < 0.001), and dorsal striatum (F_3,48_ = 11.47, *p* < 0.001) but not within the hippocampus (F_3,48_ = 1.05, *p* = 0.38), as well as a diet × sex interaction in the prefrontal cortex (F_3,56_ = 7.87, *p* < 0.001), nucleus accumbens (F_3,56_ = 22.13, *p* < 0.001), and dorsal striatum (F_3,48_ = 12.75, *p* < 0.001) (Fig. 3). A maternal HFD led to increased levels of 5-HT_2C_ receptors in the nucleus accumbens (*p* < 0.001) and dorsal striatum (*p* < 0.001) only in male offspring. On the other hand, exposure to maternal HCD during pregnancy and lactation at PND 63 increased the cortical 5-HT_2C_ receptors concentration in female offspring (*p* < 0.01). Moreover, we noted that maternal HFD did not affect the levels of 5-HT_2A_ receptors in either male or female adult offspring (Fig. 4).

## Discussion

Brain plasticity during the fetal and early postnatal period is essential for proper brain development and adaptation to a changing environment, but this also means that external factors, such as maternal diet, stress or infections, can lead to permanent changes in the central nervous system in offspring [15].

In the current study, we investigated the effect of modified maternal diets (HFD, HCD, and MD) during pregnancy and lactation on the protein level of 5-HT_2C_ receptors in the brain structures of male and female offspring. Here, we report that maternal HFD reduced the level of 5-HT_2C_ receptors in the synaptosomal fraction of the prefrontal cortex, dorsal striatum, and nucleus accumbens in adolescent male rats. On the other hand, in adult males exposed to maternal HFD, we observed significantly increased levels of 5-HT_2C_ receptors within the nucleus accumbens and dorsal striatum but not in the prefrontal cortex. Moreover, in contrast to the males exposed to maternal HFD, we did not observe any changes in the protein level of 5-HT_2C_ receptors in adolescent and adult females. It is worth emphasizing that maternal HFD reduced the 5-HT_2A_ receptor level in the prefrontal cortex in adolescent males (the same direction of changes as for 5-HT_2C_ receptors) but did not induce significant changes in other structures at any of the time points examined. Therefore, it appears that the disturbed signaling mediated by 5-HT_2C_ receptors is not cognized by 5-HT_2A_ receptors.

Although the current literature does not provide data on the impact of modified maternal nutrition during pregnancy and lactation on 5-HT_2C_ and 5-HT_2A_ receptors in the offspring brain, there are some reports indicating the effect of prolonged (15–20 weeks) feeding of adult male rodents a HFD (inducing obesity) on these receptors. Thus, Lopez-Esparza et al. [16] found a reduction in the expression of 5-HT_2C_ receptors in the hippocampus in rats, while Huang and colleagues [17] reported an increased mRNA expression of these receptors in the mouse hypothalamus and increased expression of 5-HT_2A_ receptors within the olfactory nucleus.

In the present study, for the opposite effects of maternal HFD on 5-HT_2C_ receptors in limbic structures at PNDs 28 and 63 in male rats, one should take into account that such a maternal diet also affects other elements of the 5-HT system, which could lead to the differences in protein levels of 5-HT_2C_ receptors in adolescent and adult offspring. It has been shown that maternal HFD evoked an increase in tryptophan hydroxylase 2 (TPH2, an enzyme involved in 5-HT synthesis) expression at PND 28 in mice [18] and increased mRNA of 5-HT_1A_ inhibitory autoreceptors at PND 95 in rats [19] in some brain structures, effects that could correspondingly lead to an increase or reduction of 5-HT tone, and consequently to a reduction or increase of the 5-HT_2C_ receptor expression, respectively.

At the same time, the lack of an effect of maternal HFD on 5-HT_2C_ receptors in the female offspring may be related to the importance of sex hormones, as has been suggested by Gugusheff et al. [20], who examined the effect of modified maternal diets on mu-opioid receptors in rat offspring.

In some behavioral experiments, we have also demonstrated that maternal HFD (but not HCD and MD) evokes depression-like behavior in offspring as it prolongs immobility time in the forced swimming test (FST) and induces anhedonia manifesting as a decrease in sucrose preference [9, 10]. The above observations and our present results, taken together with some preclinical findings and the results of postmortem studies of the human brain in suicide victims, could suggest the involvement of 5-HT_2C_ and 5-HT_2A_ receptors in the pathogenesis of depression [6, 21]. However, such a suggestion seems doubtful since, in contrast to changes in the expression of 5-HT_2C_ and 5-HT_2A_ receptors, maternal HFD-induced depressive-like behaviors were dependent on neither the age nor the sex of the offspring [9, 10]. Moreover, the brain regions tested did not come directly from the animals used in the behavioral experiments; therefore, we cannot directly correlate the results for individual animals.

There are also a number of preclinical and clinical reports demonstrating that maternal HFD can lead to several neurodevelopmental or other mental disorders, such as ADHD, ASD, anxiety, schizophrenia, eating disorders, and cocaine-seeking behavior [3, 4, 11]. However, again, there are no data, which may indicate that they could be related to HFD-induced changes in the expression of brain 5-HT_2C_ or 5-HT_2A_ receptors.

In contrast to HFD, other diets used in the present study during pregnancy and lactation did not evoke coherent changes in the expression of 5-HT_2C_ receptors. In fact, maternal HCD reduced their expression in the adolescent male offspring but increased it at PND 63 in female animals in the prefrontal cortex only. At the same time, maternal MD decreased the expression of the 5-HT_2C_ receptors in the prefrontal cortex of males at PND 28 but increased it in the hippocampus of males and females at PND 28. Whether these changes in 5-HT_2C_ receptors have any functional meaning remains to be elucidated.

Taken together, the present study provides evidence that modified prenatal and early postnatal diets (mainly HFD) modulate the expression of 5-HT_2C_ and 5-HT_2A_ receptors in offspring brain structures. Further studies are necessary to elucidate their functional and/or pathological importance, as well as their potential impact on the efficacy of drugs (e.g., antidepressants) for which 5-HT_2C_ and 5-HT_2A_ receptors are one of their molecular targets.

## Abbreviations

ADHD: Attention-deficit hyperactivity disorder
ASD: Autism spectrum disorder
FST: Forced swimming test
HCD: High-carbohydrate diet
HFD: High-fat diet
MD: Mixed diet
PND: Postnatal day
SD: Standard diet
5-HT: Serotonin (5-hydroxytryptamine)
